# Two-Dimensional Simulation of the Freezing Characteristics in PEMFCs during Cold Start Considering Ice Crystallization Kinetics

**DOI:** 10.3390/polym14153203

**Published:** 2022-08-05

**Authors:** Panxing Jiang, Zhigang Zhan, Di Zhang, Chenlong Wang, Heng Zhang, Mu Pan

**Affiliations:** 1 School of Automotive Engineering, Wuhan University of Technology, Wuhan 430070, China; 2 Foshan Xianhu Laboratory of the Advanced Energy Science and Technology Guangdong Laboratory, Xianhu Hydrogen Valley, Foshan 528200, China; 3 State Key Laboratory of Advanced Technology for Materials Synthesis and Processing, Wuhan University of Technology, Wuhan 430070, China

**Keywords:** cold start, PEMFC, freezing process, crystallization kinetics, MEA, ice distribution

## Abstract

Cold start is one of the major issues that hinders the commercialization of polymer electrolyte membrane fuel cells (PEMFCs). In this study, a 2D transient multi-physics model is developed to simulate the cold start processes in a PEMFC. The phase change between water vapor, liquid water, and ice in the catalyst layers (CLs), micro porous layer (MPLs), and gas diffusion layers (GDLs) is also investigated, particularly the effect of ice crystallization kinetics when supercooled liquid water changes into ice. The factors affecting the different operating conditions and structural features of the membrane electrode assembly (MEA) are investigated. The results show that when the start temperature is −20 °C or higher, ice formation is delayed and the formation rate is decreased, and supercooled liquid water permeates from the CL into the MPL. For an MEA with relatively high hydrophobicity, the water permeation rate is high. These results can enable a PEMFC to start at subzero temperatures. The effect of ice crystallization kinetics is negligible when the fuel cell is started at −30 °C or below.

## 1. Introduction

Starting at subzero temperatures is one of the main hurdles in commercializing polymer electrolyte membrane fuel cell (PEMFC). A successful cold start would require a rapid temperature rise to avoid ice formation and water build-up in the porous electrode layers. A well-controlled cold start can help mitigate or even eliminate the potential damage caused by freeze/thaw or cold-start cycles.

To reduce this possible damage and enable a successful cold start, researchers have attempted to optimize start-up strategies by removing as much water as possible by gas purging [1,2] during cell shutdown, by preheating the fuel cell to increase its temperature as quickly as possible [3,4,5,6], by utilizing different current loading modes during the start-up process [7]. Meanwhile, people also tried to improve the cold start performance of fuel cells by considering the effects of end plate [8], flow-field structure [9] and the membrane electrode assembly (MEA) material components [10]. So far, numerous experimental and modeling studies have been conducted to investigate the reasons for the damage caused to the MEA, such as cracks and pinholes on the membrane [11,12], local catalyst cracks [13], interfacial catalyst layer (CL)/membrane and CL/gas diffusion layer (GDL) delamination [14,15], loss of electrochemical surface area [16], and variation in the hydrophobicity of the GDL [17,18].

Almost all of the above-mentioned damages to the MEA are related to water in the fuel cell and are caused by freeze/thaw cycles or the cold start operation; therefore, researchers have attempted to study the water phase states, phase changes [19,20,21,22], water transport models [23,24,25], liquid and ice distribution [26,27], and other behaviors of water in fuel cells at subzero temperatures.

Interestingly, one of the states of water in the PEMFC at a subzero temperature (supercooled liquid) is attracting increasing attention from researchers. S.H. Ge et al. [28,29] developed a transparent PEFC to study liquid water conversion and ice formation during start-up from subzero temperatures; they used a silver mesh as the cathode GDL to directly observe the phase change and water transport on the surface of the CL. It was found that at a current density of 0.02 A/cm^2^ and start-up temperature of −5 °C, water in the cathode CL existed in the solid and gaseous phases. However, when start-up temperatures higher than −3 °C, water droplets were found on the CL surface, and the cold start operation was significantly prolonged. Based on this, they suggested that the freezing-point depression of water in the cathode CL is not greater than 1 ± 0.5 °C and that it plays a negligible role in the cold start theory and its applications. J. B. Ryan et al. [30,31] considered supercooled water in their theoretical modeling and defined freezing-point depression as the difference between the freezing point in a porous material and the normal freezing point of water; generally the freezing-point depression of water is only about 1 ± 0.5 °C. C.W. Park et al. [20] reported their work on the supercooling release of micro-sized water droplets on GDL surfaces with cooling. It was found that the average supercooling degrees of water droplets decreased as the size of water droplets increased from 6 μL to 15 and 30 μL on the hydrophobic GDL surface, while they increased from 6.9 K to 7.5 and 10.1 K as the PTFE coating rate of the GDL increased from 0% to 40% and 60% PTFE contents, respectively. Notably, the water on the GDL surface could remain in the liquid phase for several minutes at a supercooling degree of 7–10 °C, depending on properties, such as the PTFE content, size of the water droplet, among others. P Oberholzer et al. [32] used high-resolution dynamic in-plane neutron imaging to investigate the mechanism of water accumulation during a PEFC cold start. In their work, a condensed water phase was observed to accumulate not only in the MEA but also in the cathode GDL at −15 °C and even in the cathode gas channels at −10 °C. Y. Ishikawa et al. [19] tested a fuel cell at −10 °C; they measured the temperature of the water on the CL using thermal imaging and observed the behavior of the water using a microscope under appropriate illumination. They found that the generated water was in a supercooled state, and the diameter of the water droplets was approximately 10 μm when generated. Subsequently, the size of the droplets increased considerably, absorbing the smaller water droplets in the vicinity and, consequently, becoming larger and fewer. Furthermore, the supercooled state was maintained while these physical movements occurred. In addition, the droplets froze when they expanded to the diameter of approximately 100 μm, after which the temperature rose significantly. Generally, when water in a supercooled state begins to freeze, it emits heat of solidification, and the temperature rises to 0 °C. Y. Ishikawa et al. [33] also developed a system capable of acquiring cross-sectional visible and infrared images inside the fuel cell, and they used this system to observe the supercooled water and freezing phenomena. They found that supercooled water was generated on the GDL surface, and water froze at the interface between the GDL and MEA. Using infrared radiation imaging, it was clarified that the heat of solidification disperses at the GDL/MEA interface the moment the cell performance drops. The ice formation at the GDL/MEA interface causes air gas stoppage and consequently affects cell performance.

Recently, Y. Ishikawa et al. [34] theoretically analyzed the supercooled states of water generated below the freezing point in a PEFC and demonstrated, experimentally, that inside the CL, water can be present in the liquid state for 340 s and 70 s when the supercooling degrees are 10 °C and 20 °C, respectively. Based on the heterogeneous nucleation theory and by considering the surface wettability of the porous media in the cells, they developed a theoretical model to predict the release of supercooled states. The model successfully reproduced the supercooled state in the cell, specifically its release time, and quantitatively clarified the effect of the pore diameter and wettability on the supercooled states. T.J. Dursch et al. [35,36,37] experimentally studied the ice crystallization kinetics (ICK) of water in the GDL and CL at subzero temperatures, including the effects of the rate of temperature decrease and supercooling degree on the induction time for ice crystallization (i.e., survival time for liquid water), and the crystallization rate. Therefore, water can be a supercooled liquid in the PEMFC when it is in a subzero temperature environment, although the freezing-point depression varies in a wide range; the possible reason for this is the absence of ice nuclei. Water can be maintained in a metastable liquid state in the temperature range of −42–0 °C, and its stability depends on the probability of formation and growth of ice nuclei. The presence of the liquid state and its survival time may impact water’s movement, phase transfer, and, consequently, the ice distribution inside the cell, as well as the preheating methods and optimal control strategy for the cold start. To the best of our knowledge, only a few studies have focused on this topic, among which T.J. Dursch et al. mentioned a 1D isothermal PEMFC cold-start model in their experimental research work about the ICK in CL [37]; in the model, the ICK was considered, but both the model and the results were introduced very simply. L Yao et al. numerically investigated the cold-start behavior of PEMFCs in the presence of supercooled water [38], assuming that when the freezing probability of the supercooled liquid reaches one, the liquid water inside the cell would freeze simultaneously, and no liquid water will exist in the cell.

In the early work [15], with a 2D model, we studied the mechanical response induced by the ice formation in the MEA during a failed start up procedure of fuel cell, and the stress and strain distribution and evolution were studied; the MEA may be damaged by the stress. In this study, a 2D transient multi-physic model was developed to simulate the cold-start processes in a PEMFC; the phase change between vapor water, liquid water and ice in the CLs, MPLs and GDLs was included; particularly, the ICK was considered when super cold liquid water changes into ice. The factors of different operating conditions and MEA wettability were investigated. The following sections contain a brief introduction to the characteristics of ICK, model assumptions, details of the complete mathematical model, boundary conditions and numerical procedures, results and discussion, and finally conclusions.

## 2. Ice Crystallization Kinetics

According to the heterogeneous nucleation theory [39], critical clusters may form in supercooled water at a certain supercooling degree. The production rate of such clusters, *J* (nuclei cm^−3^s^−1^), can be expressed by

(1) $$\begin{array}{cc} J(T)= & \frac{n_{L}kT}{h}\cdot \exp\left(\frac{-\Delta g}{kT}\right)\cdot \exp\left(\frac{-\Delta G^{*}}{kT}\right)\\ = & \frac{n_{L}kT}{h}\cdot \exp\left(\frac{-\Delta g}{kT}\right)\cdot \exp\left(\frac{-16\pi \cdot \sigma^{3}\cdot T_{e}^{2}}{3k\cdot T\cdot \rho^{2}\cdot h_{cond}^{2}\cdot \Delta T^{2}}\cdot f\left(\theta \right)\right) \end{array}$$

or

(2) $$J(T)=A\cdot \exp[-\frac{B}{T{\left(\Delta T\right)}^{2}}]$$

and

(3) $$A=\frac{n_{L}kT}{h}\cdot \exp(\frac{-\Delta g}{kT})$$

(4) $$B=\frac{16\pi \cdot \sigma_{}^{3}\cdot {Τ}_{e}^{2}}{3k\cdot \rho_{}^{2}\cdot h_{cond}{}^{2}}\cdot f\left(\theta \right)$$

where *n_L_* is the number density of water molecules, *k* is the Boltzmann constant, *T* is the temperature (K), *h* is the Planck constant, Δ*g* is the activation energy of water molecules, ΔG* is the Gibbs-free energy of critical nucleus formation, σ is the surface tension of the cluster and water, *T_e_* is the melting temperature, *ρ* is the mass density of water, *h_cond_* is the latent heat of condensation, Δ*T* is the supercooling degree, and *f*(*θ*) is the energy barrier coefficient of nucleation.

A supercooled state is released when the total number of critical clusters reaches a threshold value of 1. The total number of critical clusters can be calculated by integrating the product of the water volume, *V*_0_, and the critical cluster nucleation rate [34].

(5) $$I=\int_{0}^{t0}J(T,\theta )\cdot V_{0}(t)dt$$

The time from the moment liquid water is produced to the moment it is released is called the induction time, *τ_i_*, expressed by the equation below, where *J* and *V* are constants:

(6) $$\tau_{i}=\frac{1}{J(T)V_{0}}+t_{g}$$

After the release, the liquid-to-ice conversion rate, i.e., the crystallization rate of water, $R_{i}\left(t;T\right)$, follows [36,37,40]:

(7) $$\begin{array}{cc} R_{i}\left(t;T\right)= & \partial \varphi \left(t;T\right)/\partial t\\ = & q{\left(T\right)}^{2/5}\cdot \left(1-\varphi \right)\cdot {\left(-In\left(1-\varphi \right)\right)}^{3/5} \end{array}$$

where *t* is the time elapsed after the release, φ is the ice volume fraction:

(8) $$\varphi \left(t;T\right)=1-\exp\left[-q\left(T\right)\cdot \left(t-\tau_{i}{\left(T\right)}^{5/2}\right)\right]$$

and *q*(*T*) is the crystallization rate constant (s^−2.5^) under a certain contact angle, *θ*, and supercooling degree, Δ*T*.

Therefore, the liquid converts to ice at a limited rate, which differs from the instantaneous phase change based on thermodynamics [23,24,38,41].

**Figure 1:**
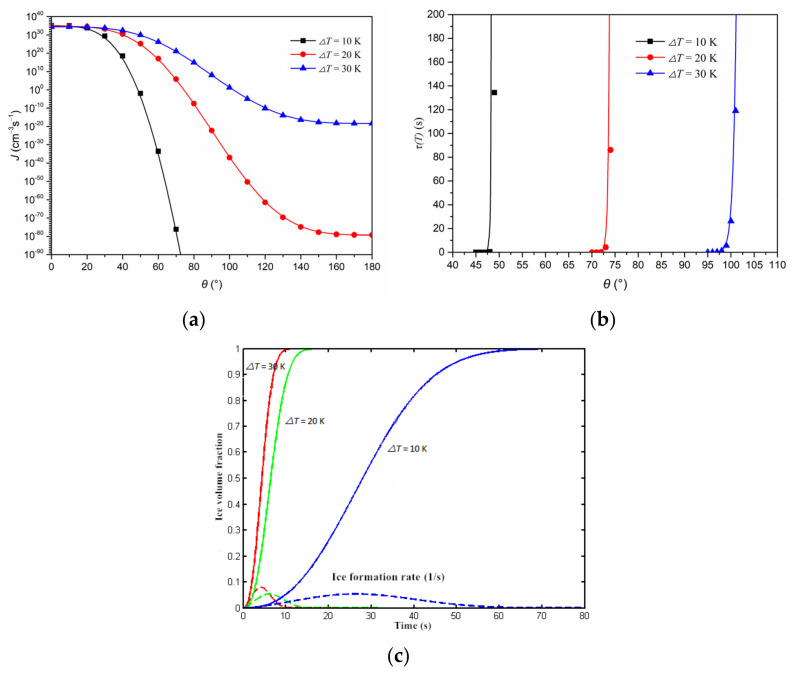
Ice crystallization characteristics in the MEA. (**a**) Crystal nucleus rate vs. contact angle. (**b**) Induction time vs. contact angle. (**c**) Ice volume fraction and ice formation rate vs. time, *θ* = 60°. The solid and dashed lines represent ice volume fraction and ice formation rate at different supercooling degree, respectively.

Theoretically, using the constants and parameters in Table 1, the relationship between the nuclei production rate, induction time, and ice crystallization rate with respect to the supercooling degree and contact angle can be computed theoretically, as shown in Figure 1.

Figure 1a,b show that, at a given supercooling degree, Δ*T*, the ice crystal nucleus production rate, *J*, decreases and the induction time, $\tau_{i}$, increases with the increase in the contact angle, *θ*; under a given *θ*, *J* decreases and $\tau_{i}$ increases as Δ*T* decreases. Figure 1c shows that after the release, the ice volume fraction, φ, increases continuously until 100% is attained; however, the ice formation rate, *R_i_*(*t*; *T*), is relatively low at the beginning and ending periods, while it is high during the middle period. Meanwhile, at a certain time and contact angle, their values increase with the supercooling degree. An implication of these trends is that the ice formation in the MEA would be suppressed at high contact angles (or on a more hydrophobic surface) [42,43]. Naturally, such ICK will affect the ice formation and its distribution inside the MEA during a cold start.

Contrarily, due to the complexity of the microstructure and the material ingredients of the MEA, it is difficult to calculate the theoretical values of *J*, *τ_i_*, and *φ* even at a given supercooling degree, Δ*T*. Therefore, in this study, the measured data from Refs. [35,36,37] will be used to calculate J, which is related to coefficients *A* and *B* in Equation (2). Meanwhile, T.J. Dursch et al. verified that for some different CL and GDL materials, ln *J* versus *T*^−1^(Δ*T*)^−2^ produces a straight line with an intercept of ln *A* and slope of −*B*, which agrees with Equation (2) [37].

## 3. Model Description

### 3.1. Geometry Model and Assumption

**Figure 2:**
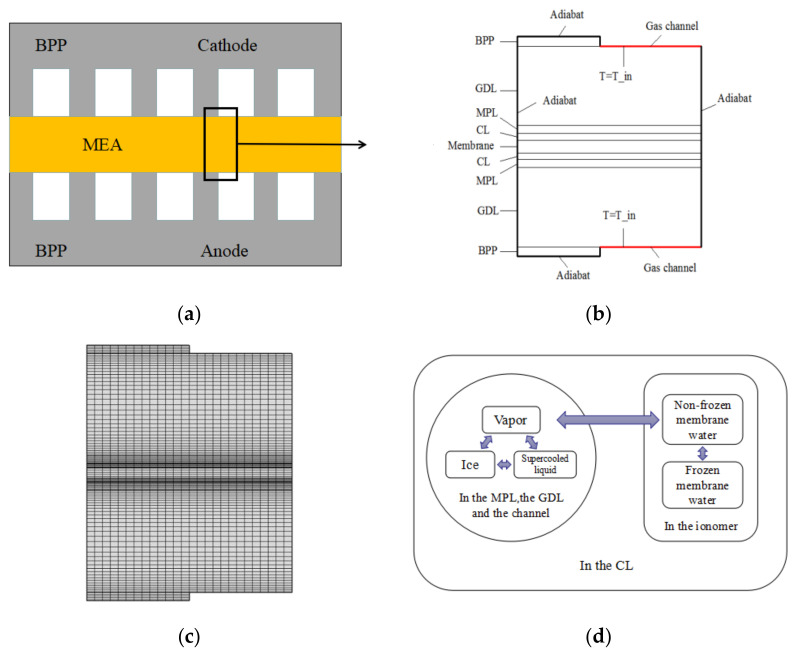
Model and water phase changes. (**a**) Geometry model; (**b**) Computational domain; (**c**) Mesh model; (**d**) Water phase changes in the PEMFC.

A 2D PEMFC model is selected to study the transport and distribution of water during a cold start, as shown in Figure 2a–c. It includes all the components, i.e., the bipolar plates (BPPs), GDLs, MPLs, CLs, and membrane. In a practical cold-start process, the air flow rate is usually much higher than that needed for the electrochemical reaction, the multi-physic field distributions have little gradient along the gas channel direction [8,44,45], a 3D structure therefore can be simplified as a 2D model; although the model of ice crystallization kinetics is based on a 3D space assumption, the thickness of the geometry of our 2D model is big enough compared to molecular size, which ensures the validity of 3D model of crystallization. Figure 2c shows the mesh model, where a grid sensitivity was performed with several levels of grid refinement, and it was determined that adequate resolution is provided by grid consisting of 26,000 elements used for this study.

At a subzero temperature, the water phases and their possible interconversions inside a PEMFC are rather complex, as shown in Figure 2d. In this study, we assume the following:

- In the MPL, GDL, and gas channel, water may exist as vapor, liquid, or ice.
- In the ionomer of the membrane, nonfrozen membrane water is present.
- In the CL, which includes both pores and ionomer, all the mentioned water phases may be present.
- When phase changes occur, there may be a supercooling degree or a superheating degree; owing to the complexity and lack of experimental data, only the ICK is considered when liquid water changes into ice, as described in the above section.

**Figure 3:**
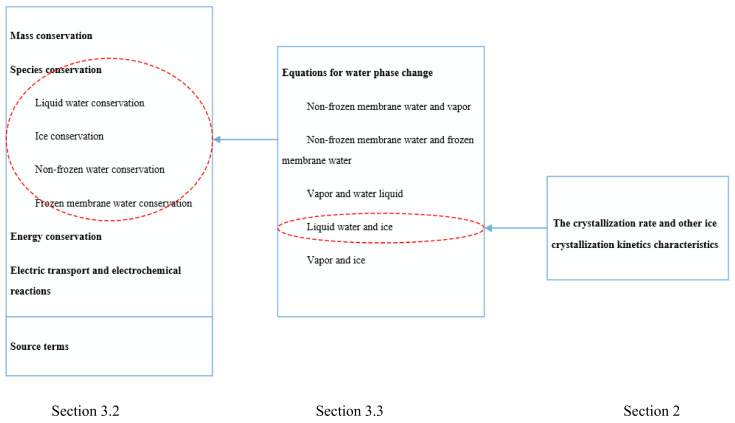
The structure of the complete mathematical model in this 2D model; the gases flowing in the gas channels and the MEA are not considered; the momentum conservation equations are not solved; the convection terms in the equations of mass conservation, species conservation, liquid water conservation, and energy conservation are omitted; conversely, the gas species of hydrogen, oxygen, nitrogen, and water vapor are considered in the present model and their transports are described by the following conservation equations [23,24,41].

Based on the above-mentioned assumptions, we developed a mathematical model. For clarity, the structure of the complete mathematical model is shown in Figure 3.

### 3.2. Conservation and Electrochemistry Equations

Mass Conservation

(9) $$\frac{\partial [\varepsilon (1-S_{ice}-S_{lq})\rho ]}{\partial t}=S_{m}$$

Species Conservation

(10) $$\frac{\partial [\varepsilon (1-S_{ice}-S_{lq})C_{i}]}{\partial t}=\nabla \cdot (D_{i}^{eff}\nabla C_{i})+S_{i}$$

(11) $$D_{i}^{eff}=D_{i}\varepsilon^{1.5}{\left(1-S_{ice}-S_{lq}\right)}^{1.5}$$

Liquid Water Conservation

The conservation equation for supercooled liquid water is given as Equation (12) in Section 3.3:

(12) $$\frac{\partial \left({\varepsilon s}_{lq}\rho_{lq}\right)}{\partial t}=\nabla \cdot \left(-\frac{K_{lq}{dp}_{c}}{\mu_{lq}{ds}_{lq}}\rho_{lq}\nabla s_{lq}\right)+S_{lq}$$

The liquid water is driven by capillary pressure to flow from a high *S_lq_* area to a low *S_lq_* area both in the cathode and anode CLs, MPLs, and GDLs.

(13) $$P_{C}=\left\{\begin{array}{l} \sigma \cos\theta {\left(\varepsilon /K_{0}\right)}^{0.5}\left[1.42\left(1-s_{lq}\right)-2.12{\left(1-s_{lq}\right)}^{2}+1.26{\left(1-s_{lq}\right)}^{3}\right]\begin{array}{cc} if & \theta <90^{o} \end{array}\\ \sigma \cos\theta {\left(\varepsilon /K_{0}\right)}^{0.5}\left[1.42s_{lq}-2.12s_{lq}{}^{2}+1.26s_{lq}{}^{3}\right]\begin{array}{cc} if & \theta >90^{o} \end{array} \end{array}\right.$$

where $K_{0}$ is the intrinsic permeability of CL, MPL, and GDL.

Ice Conservation

The conservation equation for ice is solved both in the cathode and anode CLs, MPLs, and GDLs, as follows:

(14) $$\frac{\partial \left({\varepsilon s}_{ice}\rho_{ice}\right)}{\partial t}=S_{ice}$$

Nonfrozen Water Conservation

The nonfrozen water conservation equation is solved inside the ionomer of both CLs and the membrane:

(15) $$\frac{\partial }{\partial t}\left(\frac{\rho_{m}{\omega \lambda }_{nf}}{EW}\right)=\nabla \cdot {(\omega }^{1.5}D_{nf}\nabla \lambda_{nf})+S_{nf}$$

Water Content Diffusivity

(16) $$D_{nf}=\frac{\rho_{m}}{EW}D_{w}^{m}$$

Membrane Water Diffusivity

(17) $$D_{w}^{m}=\left\{\begin{array}{cc} 3.1\times 10^{-7}\lambda_{nf}(e^{0.28\lambda }-1)e^{\left(-2346/T\right)} & 0<\lambda_{nf}\leq 3\\ 4.17\times 10^{-8}\lambda_{nf}(1+161e^{-\lambda })e^{\left(-2346/T\right)} & otherwise \end{array}\right.$$

The membrane water and vapor are in equilibrium both at the surface of the ionomers and the pores and at the surface of the CLs and the membrane, as Equation (25) shows.

Frozen Membrane Water Conservation

The frozen membrane water conservation equation is solved inside the ionomer of both the CLs and the membrane:

(18) $$\frac{\partial }{\partial t}\left(\frac{\rho_{m}\rho_{f}}{EW}\right)=S_{f}$$

The frozen and nonfrozen membrane waters can be interconverted according to Equation (28).

Energy Conservation

The conservation equation of energy is written as

(19) $$\frac{\partial }{\partial t}[(\rho C_{p}{)}^{eff}T]=\nabla \cdot (\kappa^{eff}\nabla T)+S_{T}$$

Electric Transport and Electrochemical Reactions

In both the anode and cathode CLs, the following equations are solved.

Proton Transport

(20) $$\nabla \cdot (\kappa_{ion}^{eff}\nabla \varphi_{ion})+S_{ion}=0$$

Electron Transport

(21) $$\nabla \cdot (\kappa_{ele}^{eff}\nabla \varphi_{ele})+S_{ele}=0$$

In the cathode CL, $S_{ion}=-j_{c}$, $S_{ele}=j_{c}$, and in the anode CL, $S_{ele}=-j_{a}$ and $S_{ion}=j_{a}$, where

(22) $$j_{a}=a^{eff}\cdot j_{0,a}^{ref}{\left(\frac{c_{H_{2}}}{c_{H_{2},ref}}\right)}^{1/2}(\frac{\alpha_{a}+\alpha_{c}}{RT}\cdot F\cdot \eta )$$

(23) $$j_{c}=a^{eff}\cdot j_{0,c}^{ref}(\frac{c_{O_{2}}}{c_{O_{2},ref}})\exp(-\frac{\alpha_{c}}{RT}\cdot F\cdot \eta )$$

In the anode, $\eta =\varphi_{ele}-\varphi_{ion}$, and in the cathode, $\eta =\varphi_{ele}-\varphi_{ion}-U_{0}$, where $U_{0}$ is the open-circuit potential:

(24) $$U_{0}=1.23-0.9\times 10^{-3}(T-298)$$

Due to the ice and liquid coverages, the active catalyst surface is modeled as

(25) $$a^{eff}=(1-s_{ice}-s_{lq})a$$

The source terms in the equations presented above vary locally and are summarized in Table 2 and Table 3.

### 3.3. Equations for Water Phase Change

The water produced is assumed to exist in the vapor phase, which is the nonfrozen membrane water preferentially absorbed by the ionomer until the equilibrium state is reached.

(26) $$S_{n-v}=R_{n-v}\frac{\rho_{mem}}{EW}(\lambda_{nf}-\lambda_{equil})(1-s_{lq}-s_{ice})\;\;\;\;\mathrm{kmol}\cdot m^{-3}s^{-1},$$

where *S* is the source terms (kmol m^−3^ s^−1^), *R* is the phase change rate (s^−1^), ρ is the mass density (kg m^−3^), *EW* is the equivalent weight of the proton exchange membrane (g kmol^−1^), *λ* is the water content, and *s* denotes saturation. For the subscripts, *n-v* denotes the nonfrozen membrane water to vapor, mem represents membrane, *nf* represents nonfrozen, *equil* is equilibrium, and *lq* denotes liquid water.

The equilibrium membrane water content, *λ_equil_*, (water uptake) is calculated using the correlation in [46]:

(27) $$\lambda_{equil}=\left\{\begin{array}{l} 0.043+17.81a-39.85a^{2}+36.0a^{3}\;\;if\;0\leq a\leq 1\\ 14.0+1.4(a-1)\;\;\;\;\;\;\;\;\;\;\;\;\;\;\;\;\;\;\;\;\;\;\;\;\;\;\;\;\;\;\;\;if\;1<a\leq 3 \end{array}\right.$$

where *a* is the water activity, defined as $a=\frac{X_{vp}p_{g}}{p_{sat}}+{2s}_{lq}$; *X* is the mole fraction, *p* is the pressure (pa), *vp* represents water vapor, *g* denotes the gas phase, and *sat* represents saturation.

At subzero temperatures, the maximum amount of nonfrozen water in the ionomer decreases with temperature, as was revealed by experiments [47]. Based on the experimental results, the following correlation was developed to calculate the maximum nonfrozen membrane water content, *λ_sat_*, in the ionomer before freezing [41].

(28) $$\lambda_{sat}=\left\{\begin{array}{l} 4.837\;\;if\;T<223.15K\\ {\left[-1.304+0.01479T-3.594\times 10^{-5}T^{2}\right]}^{-1}\\ >\lambda_{nf}\;\;if\;T\geq T_{N} \end{array}\right.\;\;\;if\;\;\;223.15K\;\leq \;T\;<\;T_{N}$$

The above correlation is used in the present study, which is considered as more reasonable than the correlation for room temperature or above used by many modeling works, e.g., [23,24].

Frozen and nonfrozen membrane waters can be interconverted when possible:

(29) $$S_{n-f}=\left\{\begin{array}{ll} R_{n-f}\frac{\rho_{mem}}{EW}(\lambda_{nf}-\lambda_{sat}) & if\;\lambda_{nf}\geq \lambda_{sat}\\ R_{n-f}\frac{\rho_{mem}}{EW}\lambda_{f} & if\;\lambda_{nf}<\lambda_{sat} \end{array}\right.\;\;\;\;\;\;\;\;\;\mathrm{kmol}\cdot m^{-3}s^{-1}$$

In the MEA pores, vapor and liquid water would be interconvertible when possible:

(30) $$S_{v-l}=\left\{\begin{array}{ll} \left\{\begin{array}{ll} R_{cond}\varepsilon (1-s_{lq}-s_{ice})\frac{\left(p_{g}X_{vp}-p_{sat}\right)M_{H_{2}O}}{RT} & if\;p_{g}X_{vp}\geq p_{sat}\\ R_{evap}\varepsilon s_{lq}\frac{\left(p_{g}X_{vp}-p_{sat}\right)M_{H_{2}O}}{RT} & if\;p_{g}X_{vp}<p_{sat} \end{array}\right. & if\;T\geq T_{N}\\ 0 & if\;T<T_{N} \end{array}\right.\;\;\;\;\;\;\;\mathrm{kg}\cdot m^{-3}s^{-1}$$

where *R_cond_* is the condensation rate, *R_evap_* is the evaporation rate, *R* is the universal gas constant (8.314 J mol^−1^ K^−1^), and *ε* is the bulk porosity.

At a given supercooling degree, water may exist as a liquid during the induction time, during which it would flow from the CL toward the GDL, driven by capillary pressure. The convection velocity of the liquid in the CL and GDL can be ignored. Therefore, the conservation equation for supercooled liquid water can be written as

(31) $$\frac{\partial \left({\varepsilon s}_{lq}\rho_{lq}\right)}{\partial t}=\nabla \cdot \left(-\frac{K_{lq}{dp}_{c}}{\mu_{lq}{ds}_{lq}}\rho_{lq}\nabla s_{lq}\right)+S_{lq}$$

(32) $$K_{lq}=K_{0}s_{{}_{lq}}^{4.0}{\left(1-s_{ice}\right)}^{4.0}$$

where *K* is the intrinsic permeability (m^2^) and *μ* is the dynamic viscosity (kg m^−1^ s^−1^). Although the hydraulic conductivity (*K_lq_/μ_lq_*) depends on temperature, the modeling work of Lei [38] and the experimental work of Jiao [48] demonstrated that the temperature difference inside the MEA is about 2–3 °C for a self-started fuel cell, which failed from −20 °C or −30 °C; therefore, we ignored the effect of temperature.

During the liquid water flow process, the supercooled liquid water may change to ice. According to [34,39], the supercooled liquid water is released or converted into ice (Equation (6)) when the integration of the product of water volume and critical cluster nucleation rate reaches unity; the crystallization rate or ice formation rate is expressed in Equation (7).

Therefore, the source term for the supercooled liquid-to-ice phase conversion is

(33) $$S_{l-i}=\left\{\begin{array}{ll} \left\{\begin{array}{cc} R_{i}\varepsilon s_{lq}\rho_{lq}^{} & ,\;\;t\geq \tau_{i}\\ 0 & ,\;\;t<\tau_{i} \end{array}\right. & T<T_{N}-\Delta T\\ \;-R_{m}\varepsilon s_{ice}\rho_{ice} & T\geq T_{N}-\Delta T \end{array}\right.$$

where $R_{i}$ is the ice formation rate shown in Equation (7).

Under certain conditions, vapor can be directly converted into ice:

(34) $$S_{v-i}=\left\{\begin{array}{ll} \left\{\begin{array}{ll} R_{desb}\varepsilon (1-s_{lq}-s_{ice})\frac{\left(p_{g}X_{vp}-p_{sat}\right)M_{H_{2}O}}{RT} & if\;p_{g}X_{vp}\geq p_{sat}\\ 0 & if\;p_{g}X_{vp}<p_{sat} \end{array}\right. & if\;T<T_{N}\&i\geq \tau_{i}\\ 0 & if\;T\geq T_{N} \end{array}\right.\;\;\;\;\;\;\;\mathrm{kg}\cdot m^{-3}s^{-1}$$

where *R_desb_* is the desublimation rate (s^−1^).

The geometry parameters, material properties, and electrochemical parameters used in this work are as shown in Table 4, Table 5, Table 6 and Table 7.

### 3.4. Boundary Conditions and Numerical Procedures

Both the right and the left sides of the model are symmetric; therefore, the two side walls are set as adiabatic boundaries. This cell is one of many single cells in the PEMFC stack; thus, the anode and cathode BPPs may be quasi-symmetric, and the stack may be covered by an insulating material when initiated from a subzero temperature; the anode and cathode BPPs are also set as adiabatic. The inlet gas temperature is equal to the ambient temperature, and neither the anode nor the cathode is humidified; the initial liquid water fraction is set as zero inside the cell, as a PEMFC is usually purged before the last stop when operated in an environment at subzero temperatures.

The model is solved with the commercial software, Comsol Multiphysics 5.2; the transient solver is adopted with the adaptive time step, and a minimum time step size of 10^−4^ s and a maximum time step size of 0.1 s are used. The Multifrontal Massively Parallel sparse direct Solver (MUMPS) algorithm is used to improve the convergence of the solution, and the convergence accuracy of all the variables is set with a tolerance of 10^−5^.

## 4. Results and Discussion

### 4.1. Comparison of Model Results and Experiment Data

**Figure 4:**
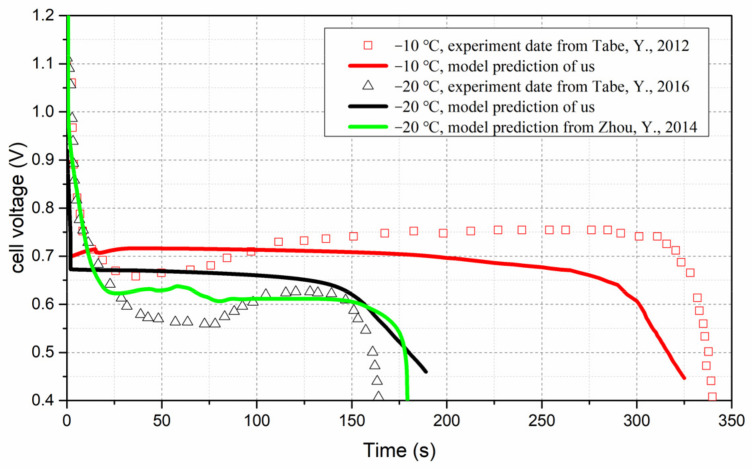
Cell voltage evolution by model prediction and by experiment, at −10 °C and −20 °C, 0.08 A/cm^2^ [44,45,49].

Figure 4 shows the cell voltage evolution curves obtained by model prediction from us and from Ref. [49], and by experiment [44,45] at −10 °C and −20 °C under 0.08 A/cm^2^. As we did not have the specifics of the structure and operating conditions of the fuel cells used in the experiment, we did not try to adjust the parameters of the simulation to make the curves fit better; however, it can be seen that the curves of our modeling and the experiment at −10 °C, and those at −20 °C, are close to each other, respectively, and their overall trends are similar. This comparison means that the model developed in this paper is reliable.

### 4.2. Comparison of the Cold Start Processes Based on ICK and Based on Thermodynamics (TD)

**Figure 5:**
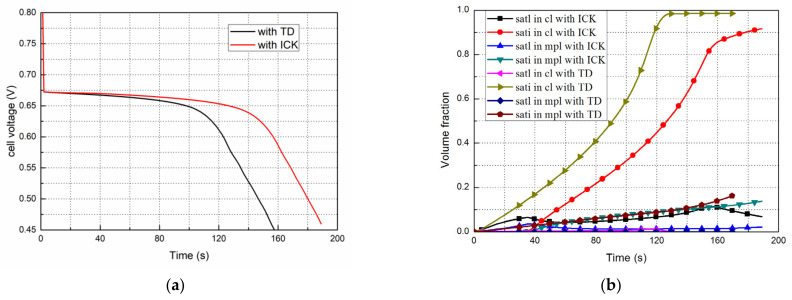
Evolution of the performances, and the liquid and ice saturations in the cathode CL and MPL when started from −20 °C at 0.08 A/cm^2^ under ICK and TD. (**a**) Performance evolution; (**b**) Liquid and ice saturation evolution.

Figure 5a shows the performance comparison of the fuel cells started from −20 °C at 0.08 A/cm^2^, based on the ICK and TD conditions, respectively.

When the ICK is considered, according to the Equation (6), the induction time of the supercooled water is 34.3 s, before which the water is in a liquid state. Furthermore, it can be seen that in the early stage of the cold start, the performances under the TD and ICK conditions are the same, which means the process of water absorption to saturation in the membrane is the same. However, due to the influence of ICK, liquid water can exist for a certain period and diffuse into the MPL. Thus, the fuel cell performance under the ICK was enhanced, and the start process was sustained for a relatively long time as compared to the operation under thermodynamic conditions only.

**Figure 6:**
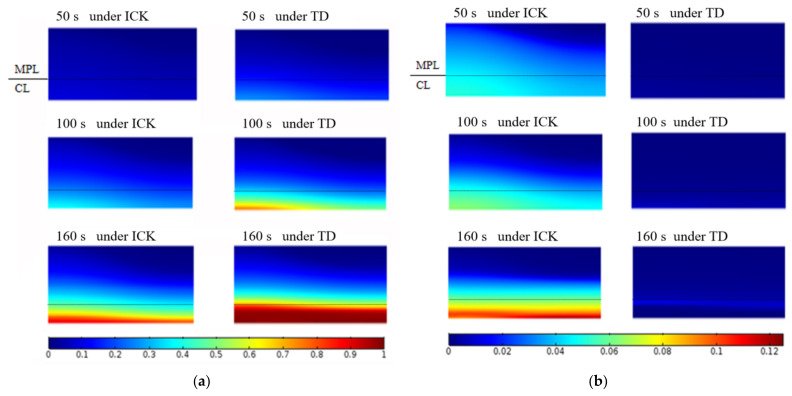
Distribution of liquid and ice in the cathode CL and MPL when started from −20 °C at 0.08 A/cm^2^ under ICK and TD. (**a**) Distribution of ice saturation fraction; (**b**) Distribution of the liquid saturation fraction.

Figure 5b shows the evolution of the average liquid and ice saturation in the CL and MPL when the fuel cell started from −20 °C at 0.08 A/cm^2^. When only the thermodynamic condition is considered, the water produced changes into ice once the fuel cell begins to work. Ice saturation increases gradually and reaches 0.85 or more at 115 s in the CL; thereafter, the pores are almost completely blocked at 160 s. During this time, the liquid saturation remained at 0 almost constantly. The volume fraction distributions of liquid water and ice in the CL and MPL of the cathode are shown in Figure 6a,b, respectively. When the ICK condition is considered, the generated water remained in the liquid state after the fuel cell began operation. The liquid saturation increased gradually until 34.3 s, after which the ice saturation increased and reached 0.85 at 160 s in the CL, during which the liquid saturation in the CL was about 0.1. Subsequently, the performance of the fuel cell gradually deteriorated, and the amount of generated water decreased. The ice saturation increased to around 0.9, and the cells stopped working at 189 s. The distributions of liquid and ice in the cathode CL and MPL are shown in Figure 6a,b, respectively. In addition, it can be observed that the liquid water content under the ICK condition is always higher than that under the TD condition. The main reasons are the following. (1) The liquid water generated during the cold start process with ICK has a certain induction time before it freezes. (2) As the volume fraction of ice increases gradually, the icing rate affected by the crystallization kinetics increases and then decreases. Compared to the case with TD, liquid water is converted to ice more slowly with ICK.

In addition, notably, compared with MPL and CL, the contents of ice and liquid water in the GDL are very small, almost zero; therefore, their volume fractions in the GDL are not added in the figure. This is mainly caused by the fact that the boundary condition is set as 30% RH air for the cathode gas channel and the GDL interface, which means that the gas flow velocity in the channel is very high, similar to the high stoichiometric ratios around 20 in the experiment of Yutaka et al. [44,45]; a large amount of water vapor is, therefore, carried away by airflow.

**Figure 7:**
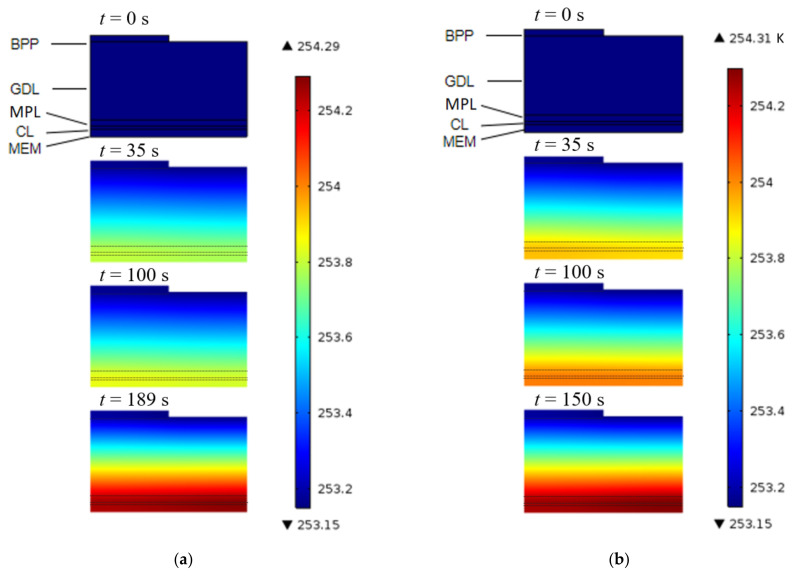
Distribution of the temperature in cathode when started from −20 °C at 0.08 A/cm^2^ under ICK and TD. (**a**) under ICK; (**b**) under TD.

Figure 7 shows the temperature contours in the cathode of the cell for the cold start from −20 °C, based on the ICK and TD conditions. It can be seen that during the initial stage of the cold start under the two conditions that the temperature at the cathode catalytic layer of the fuel cell rises rapidly. This is mainly because a large amount of ohmic heat is generated in low conductivity. Consequently, the other parts gradually heat up, although the temperature of the catalytic layer is always at the highest state. Moreover, because of the increase in the amount of membrane water until saturation, the conductivity of the fuel cell increases slowly, the heat generation of the cell decreases, and the temperature rises slowly. However, comparing the two figures, it can be seen that the temperature under the TD condition is slightly higher than that under the ICK condition, at the same time. This is mainly because the frozen process under the TD condition starts from the initiation of the cold start. When the voltage is nearly equal under the two conditions, phase change heat is generated with TD.

### 4.3. Effects of the Initial Temperature on the Start Processes

**Figure 8:**
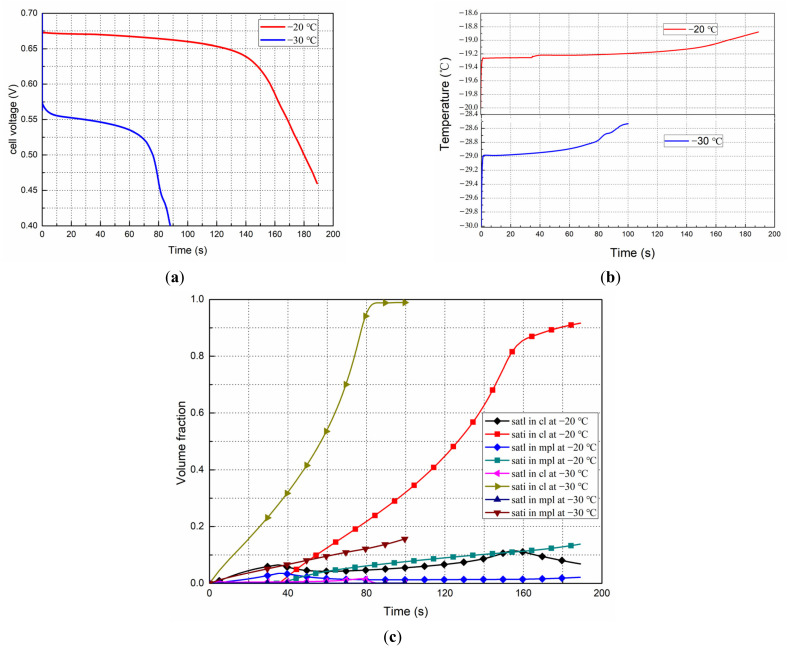
Effects of the initial start temperature on the start processes at 0.08 A/cm^2^ with initial *λ* = 6. (**a**) Performance evolution; (**b**) Cell temperature (**c**) Liquid and ice saturations in the cathode CL and MPL.

Figure 8 shows the effects of the initial temperature on the start processes. The current density is 0.08 A/cm^2^, and the initial membrane water content is *λ* = 6. The performance evolution is shown in Figure 8a. It can be seen that compared with starting up from *T* = −20 °C, it is more difficult to start from *T* = −30 °C. Under the given conditions, the temperature of the fuel cell during the two start processes increases by less than 2 °C (Figure 8b); therefore, both starts failed.

The evolution of the average liquid and ice saturation in the cathode CL and MPL with different start temperatures is shown in Figure 8c. At −20 °C and −30 °C, the saturated membrane water contents are about 8 and 6, respectively [50]. Therefore, liquid water forms immediately after the fuel cell starts. When starting from −30 °C, ice forms also almost at the same time the liquid is formed; however, when starting from −20 °C, the supercooled liquid water has an induction time of 34.3 s, and ice forms only after this period. This should be one of the main reasons why the process lasts longer when starting at −20 °C than at −30 °C.

Therefore, we can conclude that the effect of ice crystallization kinetics is negligible when the fuel cell is started from −30 °C and below; in this case, to achieve a successful cold start and reduce the possible damage to the MEA during the start process, it is recommended to preheat the fuel cells to around −15 °C firstly, which agrees with the experiment results done by the authors’ and other research groups [8,51,52].

### 4.4. Effects of Current Density on the Start Processes

**Figure 9:**
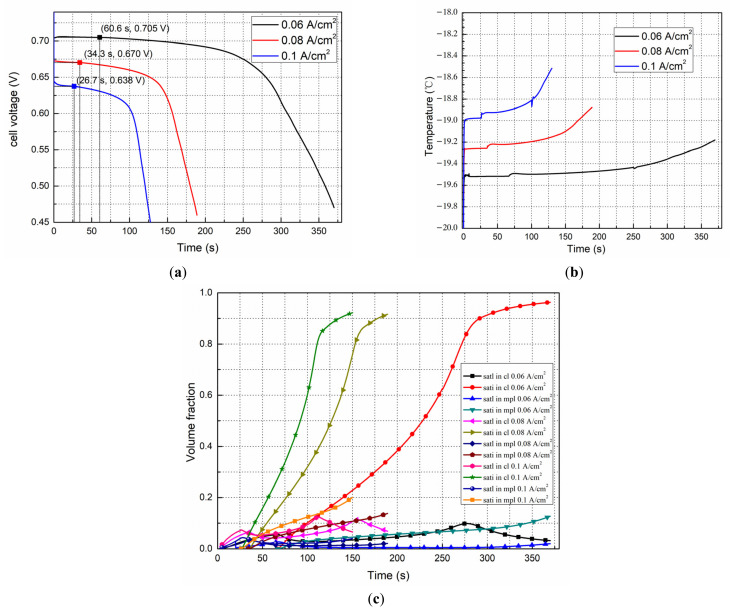
Effects of the current density on the start processes from −20 °C with initial *λ* = 6. (**a**) Performance evolution, (**b**) Temperature evolution, (**c**) Liquid and ice saturation in the cathode CL and MPL.

The evolution curves of the cell voltage with different current densities are presented in Figure 9a. The initial membrane water content is 6 with a start-up temperature, T, of −20 °C. Similar to the experimental results of Yutaka et al. [44,45], the initial cell voltage reduces and drops during the early stages of the cold start process, as the starting current density increases. During the same period, water accumulates more in the MPL. For the small current density, I = 0.06 A/cm^2^, the voltage can reach more than 0.7 V during stabilization. Correspondingly, less water is produced during the cold start process, and the formation of a critical nucleus is time-consuming. In addition, the relative accumulation of ice in the CL is reduced. However, as shown in Figure 9b, more heat will be removed from the CL, and the temperature will increase even if by <1 °C because the reaction heat is low. For a relatively high start-up current density, the water production rate is relatively high. Consequently, ice is generated earlier, which clogs the CL and causes the cold start process to fail earlier. It can be observed from Figure 9b that for the current densities of 0.06 A/cm^2^, 0.08 A/cm^2^, and 0.1 A/cm^2^, the cell temperature increases by 0.8 °C, 1.1 °C, and 1.5 °C, respectively. This also indicates that both of the cold start processes failed in the simulations. In addition, at each crystallization point corresponding to the start-up current density, the temperature of the fuel cell rises by a small degree.

Figure 9c shows the evolution of the average liquid and ice saturation in the CL and MPL when the fuel cell started from −20 °C with different start-up current densities. Due to the same starting temperature and the saturation of membrane water, liquid water will be produced almost at the same time in the three cases. However, their crystallization induction time is different. For a relatively small current density, additional time is required to accumulate liquid water and freeze it. Therefore, the corresponding cold start process will also last longer. For the current densities of I = 0.06 A/cm^2^, 0.08 A/cm^2^, and 0.1 A/cm^2^, the freezing periods are 66.6 s, 34.3 s, and 26.6 s.

### 4.5. Effects of the Initial Membrane Water Content on the Start Progress

**Figure 10:**
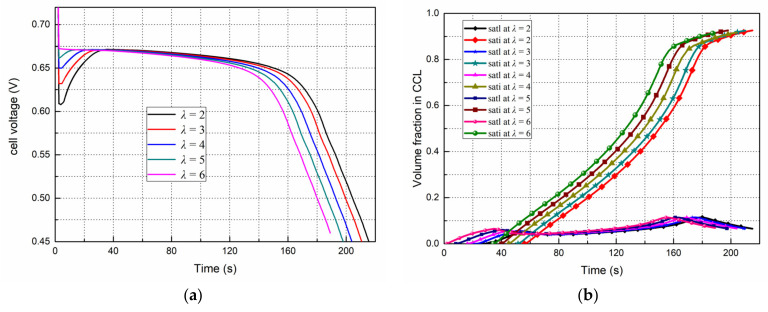
Effects of the initial membrane water content on the start processes from −20 °C, 0.08 A/cm^2^. (**a**) Performance evolution (**b**) Liquid and ice saturation in the cathode CL.

Figure 10 show the effects of different initial membrane water content, which represents the water distribution state in the fuel cell after different purging operations before it is kept in the subzero temperature environment.

The Figure 10a,b show, respectively, the evolution of voltage and liquid/ice saturation in the cathode CL with time under the conditions of −20 °C, 0.08 A/cm^2^ at different initial membrane water content (λ = 2, 3, 4, 5, 6). We can see, at the initial moment when the load is applied, the smaller the λ, the lower the proton conductivity of the electrolyte, and the bigger the voltage drop is. Meanwhile, for λ from 2 to 6, the periods before liquid water begin to appear are 27 s, 20 s, 16.2 s, 8.2 s and 1.7 s, respectively. During this period, the ionomer absorbs the water generated by the electrochemical reaction and gradually reaches saturation. Therefore, the lower initial water content mean that the ionomer has the ability to absorb more water (during this process the voltage will rise), then it takes more time for the vapor to accumulate to reach saturated vapor pressure and liquefy. As a result, the time for the supercooled water to start to freeze is prolonged, and the cold start time lasts longer.

### 4.6. Effects of the CL Contact Angle on the Start Progress

Three different CL contact angles were considered, i.e., 60°, 100°, and 140°. According to the experimental data in the literatures [35,36], we adopted A = 112.7 × 10^8^ m^−3^s^−1^, and B is set as 12.8 × 10^4^ K^3^, 40.3 × 10^4^ K^3^, and 65.65 × 10^4^ K^3^ for the contact angles, respectively, for Equation (2) to calculate the cluster production rate, J, when the fuel cell starts from −20 °C with an initial membrane water content of 6 at 0.08 A/cm^2^. The induction times calculated from Equation (6) are 13 s, 34 s, and 195 s.

**Figure 11:**
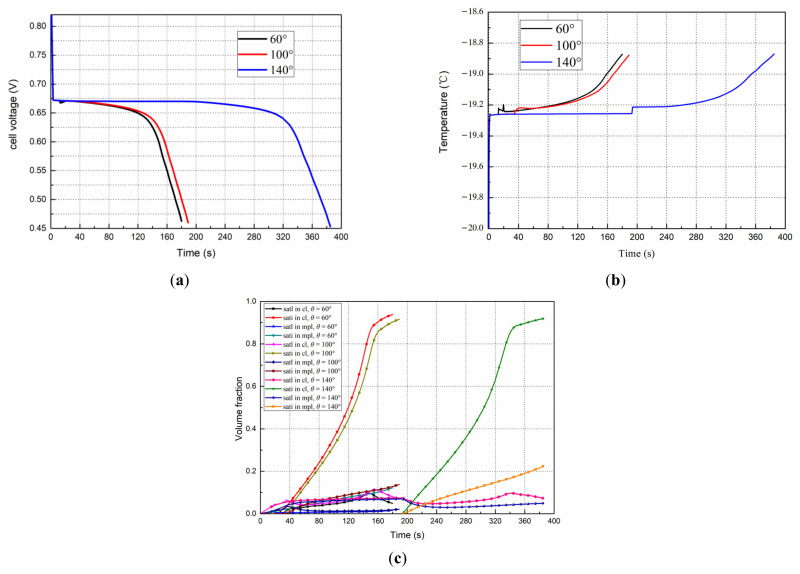
The effect of the contact angle in the CL on the start progress from −20 °C, 0.08 A/cm^2^. (**a**) Performance evolution (**b**) Temperature evolution (**c**) Liquid and ice saturation in the cathode CL and MPL.

The evolution curves of the cell voltage and the temperature for different contact angles are presented in Figure 11a,b. As the initial conditions of the cold start are the same, the voltage and temperature of the three cases are almost the same during the stable period. However, when the CL has a bigger contact angle, it is more hydrophobic, then the induction time increases; consequently, the water generated in the CL has a longer time kept as supercooled state, which means more liquid water can permeate into the MPL and GDL, the liquid and ice saturations in CL will keep at lower levels, as shown in Figure 11c. After the liquid water is released, the ice formation rate decreases at the first stage. As a result, the fuel cell running time (from the beginning to the failure moment) increases. For the relatively small contact angles of 60° and 100°, the induction times are 13 s and 34.3 s, respectively, and the difference between them is not large compared to the induction times of 195 s for 140°; the fuel cell running time is about 180 s and 190 s for 60° and 100°, respectively, and a much longer time of 385 s for 140°.

From the discussion of Section 4.4, Section 4.5, Section 4.6, we can conclude that to achieve a successful cold start and reduce the possible damage to the MEA during the start process, a lower current density should be applied to the cell to hydrate the membrane, followed by a current density as large as possible to generate more heat to increase the temperature of the cell quickly, but with a large enough air flow rate to blow the liquid water out of the cell as much as possible (as the specific heat capacity of air is low, the heat carried out by the air flow can be ignored), as done by [8].

### 4.7. Effects of the CL Bulk Porosity on the Start Progress

**Figure 12:**
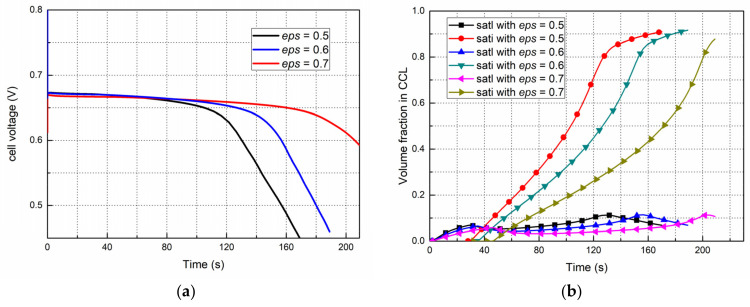
The effect of the cathode CL bulk porosity on the start progress from −20 °C, 0.08 A/cm^2^. (**a**) Performance evolution (**b**) Liquid and ice saturation in the cathode CL.

As shown in Figure 12a,b, when the porosity changes, it has no effect on the overall performance trend of the fuel cell, and the voltages at stable period are basically the same, which are about 0.67 V. From Figure 12b, liquid water appears at almost the same time, indicating that the saturation processes for the ionomer to absorb water are the same and not affected by porosity variation. Then liquid water begins to accumulate gradually, but the liquid saturation is different. When the ice saturation reaches more than 0.6, the diffusion of oxygen and the electrochemical reaction are affected by the ice block, corresponding to the time when the performance curves in Figure 12a begin to decline. However, it is obvious that the larger the porosity of the cathode cl, the longer it will take for the CL ice saturation to reach 0.6, the more slowly the fuel cell performance declines and the longer the cold start process can last.

## 5. Conclusions

In this study, a 2D transient multi-physic model was developed to simulate the cold start processes in a PEM fuel cell, the ice crystallization kinetics was considered when supercooled liquid water changes into ice, other phase change between different water states inside the MEA was included also. The model was verified by the general agreement between the predicted data and the experimental data. Thereafter, the results of the two models assuming TD and assuming ICK were analyzed and compared, the effects on the cold start processes of start temperature, current density, the wettability of the CL, and the porosity of CL were investigated. The following conclusions can be drawn.

When start temperature is −20 °C or higher, compared with models assuming TD, ice formation is delayed and the formation rate is decreased for the model assuming ICK, and more supercooled liquid water permeates from CL into MPL and GDL. Therefore, the fuel-cell performance with ICK is better, and the cold start process can be sustained for a longer time. The effect of ice crystallization kinetics is negligible when the fuel cell is started from −30 °C and below; in this case, for achieving a successfully cold start and reducing the possible damage to the MEA during the icing process, it is recommended to preheat the fuel cells to around −15 °C firstly. For a cold start with lower current density, less water is produced during the start process, and the induction time will be increased; during the same period, the amount of ice accumulated in the CL is reduced, and the cold start process can be sustained for a longer time. When the CL has a relatively large contact angle, it is more hydrophobic, and the induction time increases; consequently, more liquid water is accumulated and permeated into MPL and even GDL. As a result, the fuel-cell running time (from the beginning to the failure moment) increases.

According to the findings of this study, we propose an optimal operation control strategy for the fuel cell cold start process as follows: when the fuel cell is started from −30 °C and below, it is recommended to preheat the fuel cells to around −15 °C firstly. Then a lower current density is applied to hydrate the membrane, followed by a current density as large as possible to generate more heat to increase the temperature of the cell quickly, together with a larger air flow rate to blow the liquid water out of the cell as possible. These findings are beneficial for PEMFCs to start up from subzero temperature.

## Figures and Tables

**Table 1:** Parameters and symbols for ice crystallization kinetics.

Parameter	Symbol
A constant	*A* = 0.056 K^−1^
Number density of water molecules	*n_L_* = 3.34 × 10^22^ cm^−3^
Boltzmann constant	*k* = 1.38 × 10^−23^ J K^−1^
Planck constant	*h* = 6.63 × 10^−34^ J s
Latent heat of condensation	*h_cond_* = 334 J g^−1^
Melting temperature at 1 bar	*T_e_* = 273.15 K
The liquid thermal diffusivity	*α_L_* = 1.4 × 10^−7^ m^2^ s^−1^
Interfacial energy between cluster and water	*σ* = 3.2 × 10^−6^ J cm^−2^

**Table 2:** Source terms for water.

Domain	$S_{v}$	$S_{lq}$	$S_{i}{}_{ce}$	$S_{f}$	$S_{nf}$
GDLs and MPLs	$-S_{v-i}-S_{v-l}$	$S_{v-l}-S_{l-i}$	$S_{v-i}+S_{l-i}$	0	0
Anode CL pores	$-S_{v-i}+S_{n-v}-S_{v-l}$	$S_{v-l}-S_{l-i}$	$S_{v-i}$	0	0
Cathode CL pores	$-S_{v-i}+(S_{n-v}+(j_{c}/2F))$	$S_{v-l}-S_{l-i}$	$S_{v-i}$	0	0
CL ionomer region	$-S_{n-v}$	0	0	$S_{n-f}$	$-S_{n-v}+\nabla \cdot ((\lambda_{nf}/8F)\kappa_{ion}^{eff}\nabla \varphi_{ele})-S_{n-f}$

**Table 3:** Source terms excluding those for water.

Domain	$S_{m}$	$S_{u}{}^{*}$	$S_{i}$	$S_{ion}$	$S_{ele}$	$S_{T}$
BPs	0	0	0	0	0	${\left\|\nabla \varphi_{ele}\right\|}^{2}\kappa_{ele}^{eff}$
GDLs and MPLs	$-S_{v}$	0	0	0	0	${\left\|\nabla \varphi_{ele}\right\|}^{2}\kappa_{ele}^{eff}+h_{v-i}M_{H_{2}O}S_{v-i}+h_{v-l}M_{H_{2}O}S_{v-l}+h_{l-i}M_{H_{2}O}S_{l-i}$
Anode CL	$-(j_{a}/2F)-S_{v}$	0	$-(j_{a}/2F)$	$j_{a}$	$-j_{a}$	$\begin{array}{l} j_{a}\left|\eta_{act}\right|+{\left\|\nabla \varphi_{ele}\right\|}^{2}\kappa_{ele}^{eff}+{\left\|\nabla \varphi_{ion}\right\|}^{2}\kappa_{ion}^{eff}+h_{v-i}S_{v-i}+h_{v-l}S_{v-l}+h_{l-i}S_{l-i}\\ +(-h_{n-v}S_{n-v}+h_{n-f}S_{n-f})M_{H_{2}O} \end{array}$
Cathode CL	$-(j_{c}/4F)-S_{v}$	0	$-(j_{c}/4F)$	$-j_{c}$	$j_{c}$	$\begin{array}{l} -j_{c}T(dU_{0}/dT)+j_{c}\left|\eta_{act}\right|+{\left\|\nabla \varphi_{ele}\right\|}^{2}\kappa_{ele}^{eff}+{\left\|\nabla \varphi_{ion}\right\|}^{2}\kappa_{ion}^{eff}+h_{v-i}S_{v-i}+h_{v-l}S_{v-l}\\ +h_{l-i}S_{l-i}+(-h_{n-v}S_{n-v}+h_{n-f}S_{n-f})M_{H_{2}O} \end{array}$
Membrane	0	0	0	0	0	${\left\|\nabla \varphi_{ion}\right\|}^{2}\kappa_{ion}^{eff}+h_{n-f}S_{n-f}M_{H_{2}O}$

* In this 2D model, the gases flowing in the gas channels and the MEA are not considered, and the momentum conservation equations are not solved.

**Table 4:** Geometry parameters of the present model.

Parameter	Value
Thicknesses of the membrane, CL, MPL, GDL, and BPP	25 μm, 10 μm, 20 μm, 250 μm, and 20 μm
Widths of the membrane, CL, MPL, GDL, and BPP	500 μm, 500 μm, 500 μm, 500 μm, and 250 μm

**Table 5:** Material properties [34,41,48].

Parameter	Value
Densities of the membrane, CL, MPL, GDL, and BPP	$\rho_{mem,CL,MPL,GDL,BP}=1980;1000;1000;7800\;\;{\mathrm{kg}\;m}^{-3}$
Volume fraction of the ionomer in the CL	ω=0.25
Porosities of the CL, MPL, and GDL	ε=0.6;0.6;0.8
Contact angles of the CL, MPL, and GDL	$\theta_{CL,MPL,GDL}=60{}^{\circ};100{}^{\circ};140{}^{\circ}$
Intrinsic permeabilities of the CL, MPL, and GDL	${\left(K_{0}\right)}_{CL,MPL}=6.2\times 10^{-13};{\left(K_{0}\right)}_{GDL}=6.2\times 10^{-12}\;m^{2}$
Specific heat capacities of the membrane, CL, MPL, GDL, and BPP	${\left(C_{p}\right)}_{mem,CL,MPL,GDL,BP}=833;3300;1006;568;1580\;J\;{\mathrm{kg}}^{-1}K^{-1}$
Thermal conductivities of the membrane, CL, MPL, GDL, and BPP	$k_{mem,CL,MPL,GDL,BP}=0.95;1.0;1.0;1.0;20\;W\;m^{-1}K^{-1}$

**Table 6:** Electrochemical parameters [23,24,41,48].

Parameter	Value
Electrical conductivities of the CL, MPL, GDL, and BPP	$\kappa_{CL,MPL,GDL,BP}=300;300;300;20000\;{S\;m}^{-1}$
Ion conductivity	$\kappa_{ion}=\left({0.5139\lambda }_{nf}-0.326\right)\exp\left[1268\left(\frac{1}{303.15}-\frac{1}{T}\right)\right]\;{S\;m}^{-1}$
Effective electron conductivity and ion conductivity	$\kappa_{ion}^{eff}=\omega^{1.5}\kappa_{ion};\;\kappa_{ele}^{eff}={\left(1-\varepsilon -\omega \right)}^{1.5}\kappa_{ele}$
Electro-osmotic drag (EOD) drag coefficient	$n_{d}=\frac{2.5\lambda_{nf}}{22}$
Transfer coefficient	$\alpha_{a}=\alpha_{c}=0.5$
Volumetric reference exchange current density in the anode	$j_{0,a}^{ref}=10^{9}\exp\left[-1400\left(\frac{1}{T}-\frac{1}{353.15}\right)\right]\;{A\;m}^{-3}$
Volumetric reference exchange current density in the cathode	$j_{0,c}^{ref}=10^{4}\exp\left[-7900\left(\frac{1}{T}-\frac{1}{353.15}\right)\right]\;{A\;m}^{-3}$
Reference hydrogen and oxygen concentrations	$C_{O_{2}}^{ref}=C_{H_{2}}^{ref}=40\;{\mathrm{mol}\;m}^{-3}$
Current densities	$I=0.06\;{A/\mathrm{cm}}^{2};\;0.08\;{A/\mathrm{cm}}^{2};\;0.1\;{A/\mathrm{cm}}^{2}$
Relative humidities of the inlet gases	$Rh_{a}^{in}=0\%;\;Rh_{c}^{in}=30\%$
Inlet gas temperatures	$T_{a}^{in}=T_{c}^{in}=243.15\;K;\;253.15\;K;\;263.15\;K\;$

**Table 7:** Mass transfer parameters [41,48].

Parameter	Value
Gas dynamic viscosity in the anode and cathode	$\mu_{g}^{a}=1.53\times 10^{-5}\;{\mathrm{kg}\;m}^{-1}s^{-1};\;\mu_{g}^{c}=1.79\times 10^{-5}\;{\mathrm{kg}\;m}^{-1}s^{-1}$
Liquid water dynamic viscosity	$\mu_{lq}=2.414\times 10^{-5}\times 10^{247.8/(T-140)}\;{\mathrm{kg}\;m}^{-1}s^{-1}$
Liquid water and ice densities	$\rho_{lq}=1000\;{\mathrm{kg}\;m}^{-3};\;\rho_{ice}=920\;{\mathrm{kg}\;m}^{-3}$
Evaporation and condensation rates	$R_{evap}={1\;s}^{-1};\;R_{cond}={1\;s}^{-1}$
Fusion and melting rates	$R_{fusn}={1\;s}^{-1};\;R_{melt}={1\;s}^{-1}$
Desublimation rate	$R_{desb}={1\;s}^{-1}$
Water transfer rates	$R_{v-n,n-v}={1\;s}^{-1};\;R_{n-f\;,\;f-n}={1\;s}^{-1}$
Latent heat of fusion	$h_{fusn}=h_{n-f}=h_{l-i}=3.336\times 10^{5}\;{J\;\mathrm{kg}}^{-1}$
Latent heat of condensation	$h_{cond}=h_{v-l}=h_{v-n}=-2438.5T+3170700\;{J\;\mathrm{kg}}^{-1}$
Specific heat capacities of different gas species	${\left(C_{p}\right)}_{H_{2},O_{2},vp}=14283;\;919;\;2014\;{J\;\mathrm{kg}}^{-1}K^{-1}$
Specific heat capacities of liquid water, and ice	${\left(C_{p}\right)}_{liquid,ice}=4182;\;2050\;{J\;\mathrm{kg}}^{-1}K^{-1}$
Thermal conductivities of different gas species	$k_{H_{2},O_{2},vp}=0.1672;\;0.0246;\;0.0261\;{W\;m}^{-1}K^{-1}$
Thermal conductivities of liquid, water, and ice	$k_{liquid,ice}=0.6;\;2.3\;{W\;m}^{-1}K^{-1}$

## Data Availability

The data presented in this study are available on request from the corresponding author.

## Nomenclature

*a*	Water activity, Volume specific area (m^2^ m^−3^)
*C*	Specific heat (J g^−1^ K^−1^), concentrations (mol m^−3^)
*D*	Mass diffusivity (m^2^ s^−1^)
*EW*	Equivalent weight of the proton exchange membrane (g kmol^−1^)
*f*(*θ*)	Energy barrier coefficient of nucleation
Δ*g*	Activation energy of water molecules (J mol^−1^)
Δ*G**	Gibbs-free energy of critical-nucleus formation (J cm^−3^)
*h*	Planck constant (J s), Latent heat (J kg^−1^)
*I*	Area current density (A m^−2^)
ICK	Ice crystallization kinetics
*j*	Volume current density (A m^−3^)
*J*	Pseudo-steady-state nucleation rate (nuclei cm^−3^s^−1^)
*k*	Boltzmann constant (J K^−1^), Thermal conductivity (W m^−1^K^−1^)
*K*	Intrinsic permeability (m^2^)
*M*	Molar mass (g mol^−1^)
*n_L_*	Number density of water molecules (cm^−3^)
*n_d_*	Electro-osmotic drag (EOD) drag coefficient
*N*	Total number of water molecules
*p*	Pressure (pa)
*q*(*T*)	Crystallization rate constant (s^−2.5^)
RH	Relative humidity
*R*	Water phase change rate (s^−1^), Universal gas constant (8.314 J mol^−1^ K^−1^)
*s*	Liquid or ice saturation
*S*	Source terms (kmol m^−3^ s^−1^), or (kg m^−3^ s^−1^)
*T*	Temperature (K)
*T_e_*	Melting temperature (K)
TD	Thermodynamic
Δ*T*	Supercooling degree (K)
*u*	Velocity (m s^−1^)
*V*_0_	Liquid volume (m^3^)
*V_w_*	Total produced water in volume at moment t (m^3^)
*X*	Mole fraction
Greek letters	
*α*	Transfer coefficient
*α_L_*	The liquid thermal diffusivity (cm^2^ s^−1^)
*ε*	Bulk porosity
*ζ*	Stoichiometry ratio
*η*	Overpotential (V)
*η*_0_	Thermal growth constant
*θ*	Contact angle (°)
*κ*	Electrical conductivity
*κ_ion_*	Ionic conductivity
*μ*	Dynamic viscosity (kg m^−1^ s^−1^)
*ρ*	Mass density (kg m^−3^)
*τ_i_*	Induction time (s)
*τ_g_*	Time for nuclei grow to an instrument-detectable size (s)
*σ*	Surface tension (J cm^−2^)
*φ*	Ice volume fraction, Electrical potential (v)
*ω*	Volume fraction of ionomer in catalyst layer
*λ*	Water content
Subscripts and superscripts	
a	Anode
BP	Bipolar plate
c	Cathode
cl	Cluster and water
CL	Catalyst layer
cond	Condensation
desb	Desublimation
eff	Effective
ele	Electronic
equil	Equilibrium
evap	Evaporation
f	Frozen
fusn	Fusion
FPD	Freezing point depression
g	Gas phase
GDL	Gas diffusion layer
H_2_	Hydrogen
i	The ith species
ice	Ice
in	Inlet
ion	Ionic
lq	Liquid water
m	Mass, for source term
melt	Melting
mem	Membrane
MPL	Micro-porous Layer
N	Normal condition
nf	Nonfrozen
O_2_	Oxygen
out	Outlet
ref	Reference state
sat	Saturation
vp	Water vapor
l-i	Liquid water to ice, vice versa
n-f	Nonfrozen membrane water to frozen membrane water, vice versa
n-v	Nonfrozen membrane water to vapor, vice versa
v-i	Vapor to ice, vice versa
v-l	Vapor to water liquid
